# Neural mechanisms underlying decisions to express or suppress emotions

**DOI:** 10.1093/scan/nsag068

**Published:** 2026-08-07

**Authors:** Yoko Mano, Koki Nakaya, Hidetsugu Komeda, Asako Toyama, Haruaki Fukuda, Yuri Miyamoto, Shinobu Kitayama, Shinsuke Suzuki

**Affiliations:** Brain Research Center, Hitotsubashi Institute for Advanced Study (HIAS-BRC), Hitotsubashi University, Kunitachi, Tokyo, 186-8601, Japan; Graduate School of Social Data Science, Hitotsubashi University, Kunitachi, Tokyo, 186-8601, Japan; College of Education, Psychology and Human Studies, Aoyama Gakuin University, Shibuya-ku, Tokyo, 150-8366, Japan; Hitotsubashi Institute for Advanced Study (HIAS), Hitotsubashi University, Kunitachi, Tokyo, 186-8601, Japan; Brain Research Center, Hitotsubashi Institute for Advanced Study (HIAS-BRC), Hitotsubashi University, Kunitachi, Tokyo, 186-8601, Japan; Graduate School of Social Data Science, Hitotsubashi University, Kunitachi, Tokyo, 186-8601, Japan; Brain Research Center, Hitotsubashi Institute for Advanced Study (HIAS-BRC), Hitotsubashi University, Kunitachi, Tokyo, 186-8601, Japan; Graduate School of Social Sciences, Hitotsubashi University, Kunitachi, Tokyo, 186-8601, Japan; Department of Psychology, University of Michigan, Ann Arbor, MI 48109, United States; Brain Research Center, Hitotsubashi Institute for Advanced Study (HIAS-BRC), Hitotsubashi University, Kunitachi, Tokyo, 186-8601, Japan; Graduate School of Social Data Science, Hitotsubashi University, Kunitachi, Tokyo, 186-8601, Japan; Centre for Brain, Mind and Markets, The University of Melbourne, Parkville, VIC 3010, Australia

**Keywords:** emotional expression, decision-making, social cognition, mentalizing, fMRI

## Abstract

Emotional expressions play a central role in social communication. The dominant view assumes that emotional expression is a spontaneous consequence of affect. In everyday social life, however, individuals often suppress internally generated emotions. Here, we investigated the behavioral and neural mechanisms underlying decisions about emotional expression, based on the view that emotional expression constitutes a form of decision-making involving evaluation of social context, anticipation of interpersonal consequences, and consideration of social norms. Using functional magnetic resonance imaging, we scanned 40 Japanese young adults (22 males, 18 females) while they decided whether to express or suppress their emotions in response to emotionally evocative scenarios. We found that decisions to express emotion were positively modulated by perceived valence and arousal. Valence was associated with activity in the inferior frontal cortex and middle temporal gyrus, whereas arousal was associated with activity in the fusiform and parahippocampal gyri. Moreover, decisions to withhold emotional expression were associated with increased activity in regions implicated in mentalizing and cognitive control, including the right temporoparietal junction and dorsolateral prefrontal cortex. Together, these findings suggest that emotional expression reflects a decision-making process integrating affective and contextual information within neural systems supporting social cognition and cognitive control.

## Introduction

Emotional expressions play a central role in social communication, allowing individuals to convey internal states, intentions, and motivations to others (Keltner and Haidt 1999). Through facial expressions, vocal cues, and bodily gestures, these signals facilitate social coordination and interpersonal understanding (Ekman 1992). For example, individuals may smile to signal friendliness, modulate their tone of voice to convey politeness, or adjust their posture and gestures to express confidence.

Critically, emotional responses are not always overtly expressed. People often suppress the expression of internally generated emotions. For instance, individuals may suppress positive emotions in situations where others are experiencing negative outcomes or conceal negative emotions in socially expected positive contexts such as celebrations or achievements. Emotional expression is therefore not merely a direct consequence of affective experience. It involves a decision-making process in which individuals determine whether their internal states should be communicated. Although previous research has elucidated how emotions are generated and regulated (Gross 1998, 2002, Buhle *et al.* 2014, Barrett 2017), little is known about how individuals decide whether to express or suppress their emotional responses.

Affective experiences are commonly characterized along two core dimensions: “valence” and “arousal” (Russell 1980, Barrett and Russell 1999, Satpute and Lindquist 2019, Lockwood *et al.* 2020). These dimensions provide a parsimonious framework for describing emotional states across a wide range of contexts. Previous research has demonstrated that valence and arousal jointly shape emotional experience and guide behavior across cognitive and social domains, including attention, memory, and decision-making (Pessoa 2008, Kuppens *et al.* 2012, 2017). These findings raise the possibility that decisions about emotional expression may likewise depend on the integration of valence and arousal.

Decisions about whether to express or suppress emotional responses require individuals to evaluate the social context, anticipate interpersonal consequences, and consider social norms. Such decision-making may involve interactions between automatic and controlled processes. Dual-process models of social cognition propose that social understanding emerges from the interplay between fast, automatic processes and slower, controlled reasoning (Lieberman *et al.* 2007, Lockwood *et al.* 2020). Emotional reactions may arise automatically at first, whereas decisions about whether to express them may depend more on controlled processes that integrate social context and internal goals. In this context, affective dimensions such as valence and arousal may provide key inputs to these decision processes, shaping whether emotions are expressed or suppressed.

As emotional expressions shape how others interpret and respond to an individual, deciding whether to express emotion likely requires simulating others’ perspectives and evaluating potential social consequences. The mentalizing network, including the temporo-parietal junction (TPJ) and medial prefrontal cortex (mPFC), is known to support reasoning about others’ mental states (Saxe and Kanwisher 2003, Saxe and Wexler 2005, Van Overwalle 2009, Lockwood *et al.* 2020, Schurz *et al.* 2021). In particular, the TPJ has been consistently implicated in perspective-taking and belief reasoning (Frith and Frith 2006, Buergi *et al.* 2026). Prior work has shown that narrative-based perspective-taking recruits the TPJ even in the absence of explicit social interaction (Mano *et al.* 2009, Miao *et al.* 2026) and that narrative comprehension engages partially dissociable processes involved in estimating protagonists’ emotions and evaluating readers’ own emotional responses (Komeda *et al.* 2009, Eekhof *et al.* 2022). Complementing this perspective, recent behavioral evidence suggests that emotion-related vocabulary knowledge contributes to reading comprehension, particularly for complex and narrative texts (Sabag-Shushan *et al.* 2025), highlighting the role of affect-related semantic representations in constructing meaning. Together, these findings suggest that mentalizing-related brain regions may contribute to decision-making about emotional expression.

Individual differences in emotional awareness may further shape these processes. Alexithymia, characterized by difficulties in identifying and describing emotions (Bagby *et al.* 1994, Taylor *et al.* 2003, Bird and Cook 2013, Smith *et al.* 2017), has been associated with altered emotional and social processing. Individuals with higher alexithymia may have reduced access to internal emotional states and may therefore rely more strongly on controlled social inference processes when making emotional expression decisions. In addition to alexithymia, individual differences in self-construal have been proposed as important determinants of how individuals regulate emotional expression. Self-construal refers to the way individuals define themselves in relation to others, typically characterized along independent and interdependent dimensions (Markus and Kitayama 1991, Singelis 1994). Individuals with a more interdependent self-construal tend to prioritize social harmony and may regulate emotional expressions to align with social expectations, whereas those with a more independent self-construal may place greater emphasis on internal states.

The present study investigated the behavioral and neural mechanisms underlying decisions about emotional expression. Specifically, we examined whether the perceived valence and arousal of a situation modulate decisions to express emotions and whether this modulation varies across participants as a function of alexithymia and self-construal. Moreover, we searched for brain regions that support these decision processes and their individual variability. In our experiment, participants decided whether to express or suppress their emotions in response to emotionally evocative scenarios while undergoing functional magnetic resonance imaging (fMRI). We found that these decisions were shaped by the perceived valence and arousal of the scenarios, which were associated with activity in distributed cortical regions related to affective valuation (e.g. inferior frontal and temporal regions) and perceptual processing (e.g. fusiform and parahippocampal regions), respectively. Furthermore, decisions to suppress emotional expression recruited brain regions associated with mentalizing and cognitive control, including the TPJ and dorsolateral prefrontal cortex (Saxe and Kanwisher 2003, Ochsner and Gross 2005, Morawetz *et al.* 2020, Schurz *et al.* 2021).

## Materials and Methods

The study was approved by the institutional review board of Hitotsubashi University (Approval No. 2025C026) and conducted in accordance with the Declaration of Helsinki. The study design was not preregistered.

### Participants

A total of 46 healthy Japanese young adults participated in the experiment. Participants were recruited from local universities and surrounding communities in the Tokyo Metropolitan area. All participants provided written informed consent prior to participation. Six participants were excluded based on predefined criteria: (i) incomplete behavioral data due to failure to complete the task, (ii) interruption of the scanning session due to MRI scanner malfunction, or (iii) a self-reported history of psychiatric disorders. The final sample consisted of 40 right-handed healthy adults (22 males, 18 females; mean age = 21.1 years, SD = 2.2, range = 18–26 years). Participant characteristics are summarized in Table 1. All participants had normal or corrected-to-normal vision. Participants received monetary compensation of 6000 Japanese Yen for their participation.

The sample size of 40 was determined *a priori* based on effect-size estimates obtained from a previous study using a similar experimental paradigm (Ohnishi *et al.* 2025). Specifically, we estimated the within-subject effect size (Cohen’s *d *= 0.414) using the minimum effect size for the contrast between the Social and Control conditions in the dmPFC, TPJ, and temporal pole during the Theory-of-Mind task reported by Ohnishi *et al.* (2025). Assuming *α* = .05 and a desired statistical power of .80, the estimated required sample size for a one-sample *t*-test was approximately 38 participants. Therefore, we targeted the sample size of 40 participants to allow for potential exclusions.

### Experimental task

**Figure 1:**
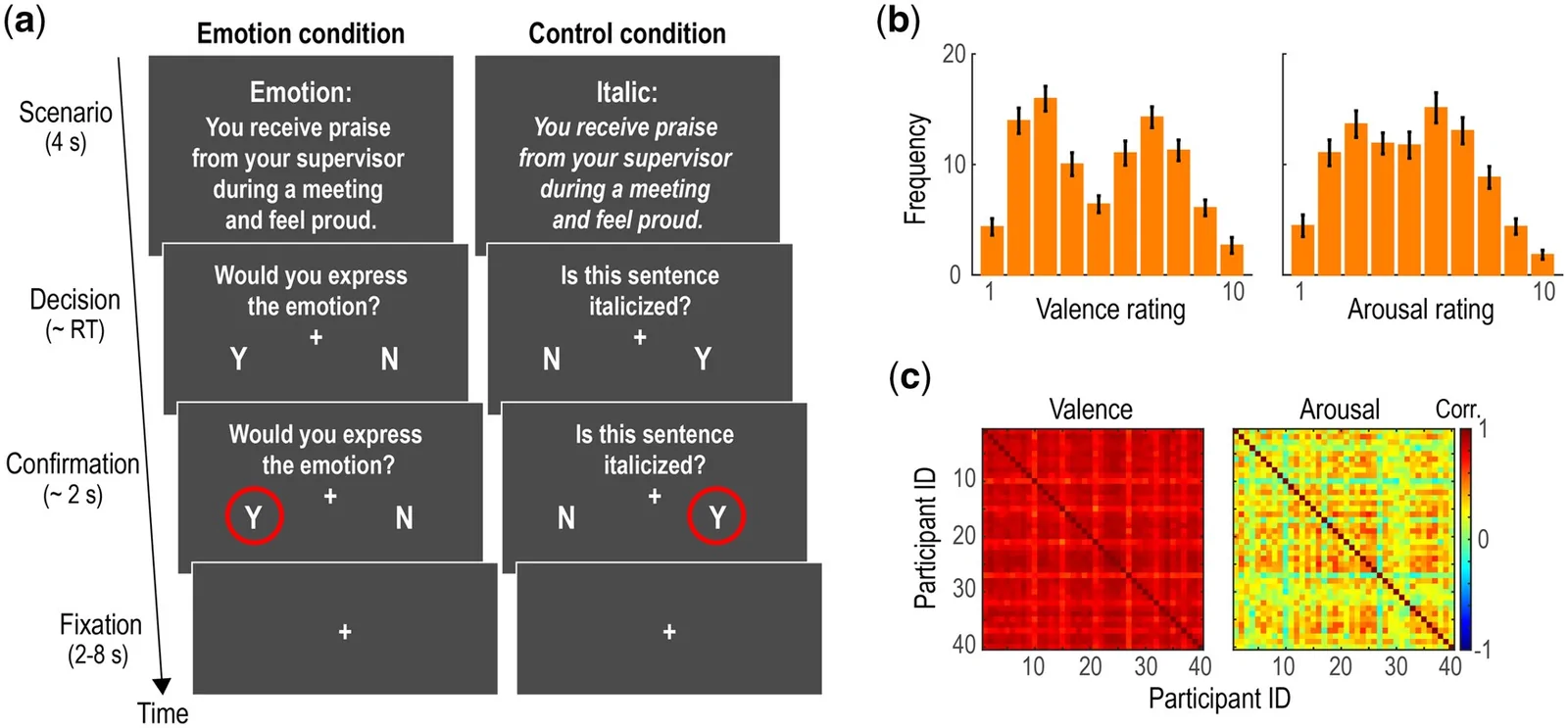
Experimental task and basic behavior. (a) Task structure. In the Emotion condition, participants decided whether they would express the emotion elicited by the scenario. In the Control condition, participants judged whether the sentence was presented in italic font. The positions of Yes (Y) and No (N) options were randomized across trials. (b) Distribution of the post-scan ratings (mean ± SEM across participants). Histograms show the distribution of valence and arousal ratings across all stimuli. (c) Across-participant similarity of the ratings. Pairwise correlations between participants are shown for valence and arousal ratings.

Participants performed an emotional expression decision task during fMRI scanning (Fig. 1a). On each trial, they were presented with a scenario sentence describing an emotional situation. Depending on the instruction cue presented above the sentence, participants performed one of two judgments. In the Emotion condition, indicated by the label “Emotion,” they judged whether they would express the emotion elicited by the scenario. In the Control condition, indicated by the label “Italic,” they judged whether the sentence was presented in italic font.

Each trial consisted of three phases: scenario presentation (4 s), decision phase (2 s), and a jittered fixation interval (2–8 s). During the decision phase, participants responded using two buttons (Yes/No). The spatial positions of the response options (left/right) were randomized across trials to dissociate decision-related processes from motor responses and spatial attention. When a response was made, the selected option was highlighted with a red circle.

Before scanning, participants were trained to understand that the instruction cue (“Emotion” or “Italic”) specified the required judgment, allowing condition-specific processing during sentence reading. In the Emotion condition, they were instructed to read the scenario, imagine themselves in the situation, evaluate the emotional response they would experience, and decide whether they would express that emotion to others. The scenario sentences were designed to cover a range of emotional situations varying in valence and arousal [see Table S1 (see online supplementary material for a color version of this table) for the full set of stimuli]. In the Control condition, participants judged whether the entire sentence was presented in italic font using the same response format.

The task consisted of four sessions of 48 trials each, yielding a total of 192 trials. Emotion and Control trials were randomly intermixed within each session. The same set of 96 scenario sentences was used across both conditions, such that each sentence appeared once in the Emotion condition and once in the Control condition in random order. In the Control condition, italicized and non-italicized sentences were presented in equal proportion.

### Post-scan ratings

After the scanning session, participants rated each of the 96 scenario sentences used in the fMRI task in terms of valence and arousal. For arousal, participants were asked: “Please indicate how emotionally aroused you would feel on a 10-point scale, where 1 indicates calm (low arousal) and 10 indicates aroused (high arousal).”

Participants also completed questionnaires assessing self-construal and alexithymia.  Self-construal was measured using a standard self-construal scale (SCS) (Singelis 1994), which provides independent and interdependent subscale scores. The self-construal difference score (ΔSC) was calculated as the interdependent minus independent self-construal score, with higher values indicating relatively stronger interdependent self-construal. Alexithymia was assessed using the 20-item Toronto Alexithymia Scale (TAS-20; Taylor *et al.* 2003), rated on a 7-point Likert scale and comprising three subscales: difficulty identifying feelings (DIF), difficulty describing feelings (DDF), and externally oriented thinking (EOT).

### Behavioral data analysis

Trials without a button response (missed trials) were excluded from all behavioral analyses.

We examined effects of perceived valence and arousal of the scenarios on the decisions about whether to express their emotions using the following generalized linear mixed-effects model (GLMM): logit(P(Yes)) ∼ 1 + *V *+* A* + (1 + *V *+* A* | Participant), where *V* and *A* denote *z*-standardized valence and arousal ratings, respectively.

We also assessed whether and how the effects of valence and arousal were moderated by self-construal and alexithymia. To this end, we included scores of alexithymia (TAS-20) and self-construal (SC) questionnaires in the GLMM: logit(P(Yes)) ∼ 1 + *V *+* A* + *Q_A_* + *Q_S_* + *V* × *Q_A_* + *V* × *Q_S_* + *A* × *Q_A_* + *A* × *Q_S_* + (1 + *V *+* A* | Participant), where *Q_A_* and *Q_S_* denote the TAS-20 and ΔSC scores, respectively.

As an exploratory analysis, we tested potential nonlinear effects of valence and arousal using the GLMM: logit(P(Yes)) ∼ 1 + *V *+* A* + *V*^2^ + *A*^2^ + (1 + *V *+* A* + *V*^2^ + *A*^2^ | Participant), where *V*^2^ and *A*^2^ indicate the quadratic effects. Furthermore, we examined whether emotional expression decisions varied across different types of social contexts: i.e. relational and individual scenarios (Table S1, see online supplementary material for a color version of this table): logit(P(Yes)) ∼ 1 + *V *+* A* + *C* + (1 + *V *+* A* + *C* | Participant), where *C* denotes the context (1 for the relational, and 0 for the individual scenarios).

We also examined the effects of “engagement” on decisions about emotional expression, based on the idea that engagement constitutes another dimension of emotion in addition to valence and arousal (Kitayama *et al.* 2000). In this additional exploratory analysis, we categorized each scenario as “engaged” or “disengaged” according to the target emotion embedded in the scenario, based on the theoretical classification proposed by Kitayama *et al.* (2000). The effects were estimated using the following GLMM analysis: logit(P(Yes)) ∼ 1 *+* *V + A + E + V × E + A × E +* (1 *+* *V + A + E + V × E + A × E | Participant*), where *E* denotes engagement (engaged = 1, disengaged = −1).

### MRI data acquisition

MRI data were acquired using a 3T Siemens MAGNETOM Prisma scanner (Siemens, Erlangen, Germany) at the HIAS Brain Research Center, Hitotsubashi University, Tokyo, Japan, equipped with a 32-channel head coil. Functional images were obtained using a multiband T2*-weighted gradient-echo echo-planar imaging (EPI) sequence (multiband factor = 6) sensitive to blood oxygenation level-dependent (BOLD) contrast. Acquisition parameters were as follows: repetition time (TR) = 800 ms, echo time (TE) = 34.4 ms, flip angle = 52°, matrix size = 86 × 86, slice thickness = 2.4 mm with no gap, and whole-brain coverage. High-resolution anatomical images were acquired using a T1-weighted magnetization-prepared rapid gradient-echo (MPRAGE) sequence (TR = 2300 ms, TE = 2.32 ms, TI = 900 ms, flip angle = 8°, voxel size = 0.9 × 0.9 × 0.9 mm, matrix size = 256 × 256).

### fMRI preprocessing

fMRI data preprocessing was performed using fMRIPrep 23.2.1 (Esteban *et al.* 2019), which is based on Nipype 1.8.6. The fMRIPrep pipeline included intensity non-uniformity correction, skull stripping, head motion correction, susceptibility distortion correction using fieldmaps, co-registration of functional and anatomical images, and spatial normalization to the MNI152NLin2009cAsym template. Following fMRIPrep preprocessing, spatial smoothing was applied using SPM25 (Wellcome Centre for Human Neuroimaging, London, UK), implemented in MATLAB. An isotropic Gaussian kernel with a full width at half maximum (FWHM) of 6 mm was used. All normalized images were visually inspected to ensure data quality and to identify potential artifacts. Detailed preprocessing procedures are provided in the Supplementary Material.

### fMRI data analysis

We used the standard general linear model (GLM) approach implemented in SPM25 (Wellcome Centre for Human Neuroimaging, London, UK) to identify brain regions involved in representing perceived valence and arousal and in decisions to suppress emotional expression.

The GLM for each participant included the following regressors: (i) sentence presentation in the Emotion condition (duration = 4 s), (ii) sentence presentation in the Control condition (duration = 4 s), (iii) decision of Yes in the Emotion condition (duration = reaction time (RT): time from decision onset to button press), (iv) decision of No in the Emotion condition (duration = RT), (v) decision of Yes in the Control condition (duration = RT), (vi) decision of No in the Control condition (duration = RT), (vii) button responses (duration = 0 s), and (viii) miss trials (duration = 4 s). Valence and arousal ratings (z-standardized within each participant) were included as parametric modulators of the sentence-presentation regressor in the Emotion condition without orthogonalization. All regressors were convolved with the canonical hemodynamic response function (HRF). Six head-motion parameters were included as nuisance regressors.

We defined three contrasts of interest: perceived valence (i.e. a parametric modulator during the sentence presentation in the Emotion condition) and arousal (i.e. the other parametric modulator), as well as the comparison between the No and Yes decisions (No > Yes) in the Emotion condition. For each participant, contrast images were estimated at every voxel across the whole brain and then submitted to a random-effects group-level analysis.

Whole-brain statistical inference was conducted using a cluster-based correction approach. First, a voxel-level threshold of *P* < .001 (uncorrected) was applied to define clusters. Statistical significance was then assessed at the cluster level using family-wise error (FWE) correction at *P* < .05.

Regions of interest (ROIs) were defined based on the peak coordinates identified in the whole-brain contrast of decisions to suppress emotional expression (No > Yes in the Emotion condition). Two spherical ROIs (6-mm radius) were centered on peak coordinates in the right temporoparietal junction (rTPJ; MNI: 60, −58, 35) and right dorsolateral prefrontal cortex (dlPFC; MNI: 48, 21, 44).

We then performed across-participant correlation analyses between ROI activity and two questionnaire-derived measures: total TAS-20 score and the difference between the independent and interdependent subscale scores of the SCS. To account for multiple comparisons, *P* values were corrected using the Bonferroni method across the total number of tests (2 ROIs × 2 questionnaire-derived measures: i.e. four comparisons).

## Results

### Basic behavior

Participants performed the task appropriately. The percentage of missed responses was 1.64% ± 0.38% (mean ± SEM across participants) in the Emotion condition and 1.02% ± 0.25% in the Control condition. In the Emotion condition, participants chose to express their emotions on 55.5% ± 2.4% of the valid trials (mean ± SEM). In the Control condition, participants correctly judged whether the sentence stimulus was presented in italic font on 96.88% ± 0.41% of the trials.

Post-scanning ratings of valence and arousal for each of the 96 sentence stimuli covered reasonable ranges (Fig. 1b). Although valence ratings showed little variability across participants (Fig. 1c, *left*), arousal ratings varied substantially across participants (Fig. 1c, *right*). Importantly, valence and arousal ratings did not share much variance within participants (*r *= −0.02 ± 0.05, mean ± SEM across participants), enabling us to examine their dissociable effects on behavior and neural activity.

### Decision-making about emotion expression

**Figure 2:**
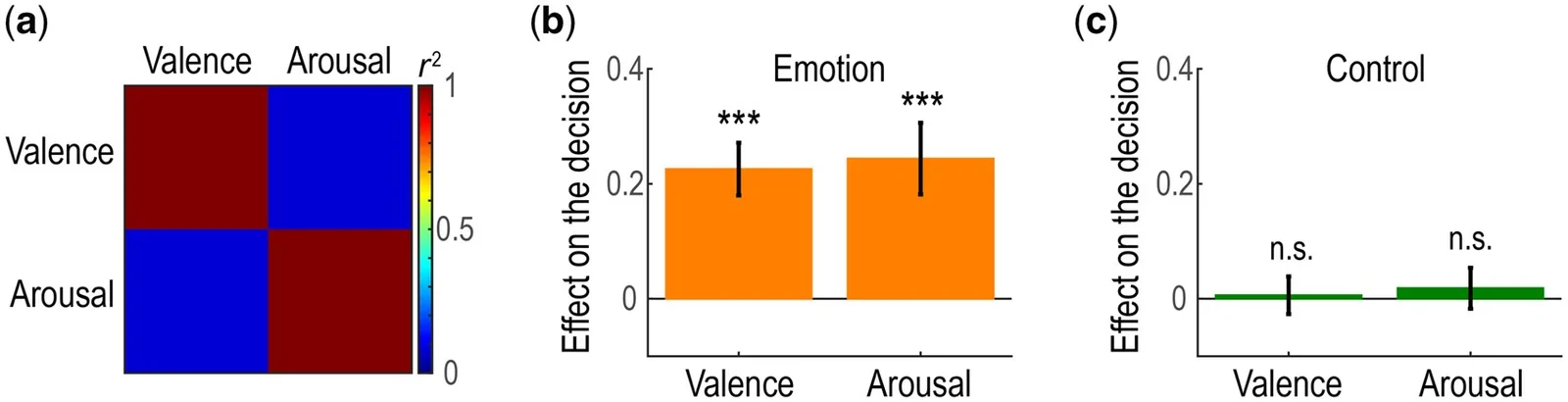
Effects of valence and arousal on emotional expression decisions. (a) Within-participant shared variance between valence and arousal ratings. Shared variance was quantified as the squared correlation coefficient (*r*^2^) and then averaged across participants. (b) Effects of valence and arousal on the decisions to express the emotion (i.e. Yes) in the Emotion condition (mean ± SEM). ***P < .001. (c) Effects of valence and arousal on judgments of whether the sentence was presented in italic font in the Control condition. n.s., non-significant. The format is the same as in (b).

Participants’ decisions about whether to express their emotions were modulated by the perceived valence and arousal of the scenarios in the Emotion condition. Specifically, both valence and arousal were positively associated with the likelihood of emotional expression (Fig. 2b; *β*  =  0.227, *SE *= 0.046, *z *= 4.94, *P* < .001 for valence; and *β*  =  0.246, *SE *= 0.062, *z *= 3.96, *P* < .001 for arousal). As expected, in the Control condition, neither perceived valence nor arousal was associated with participants’ judgments of whether the sentence was presented in italic font (Fig. 2c; *β*  =  0.005, *SE *= 0.033, *z *= 0.17, *P* = .868 for valence; and *β*  =  0.016, *SE *= 0.036, *z *= 0.44, *P* = .661 for arousal). These results suggest that participants were more likely to express their emotions in scenarios perceived as more emotionally positive and more arousing.

We next examined whether these effects were moderated by individual differences in alexithymia and self-construal difference score (ΔSC). No significant moderation effects were observed in this additional analysis (alexithymia: *β*  =  0.047, *SE *= 0.114, *z *= 0.41, *P* = .680; ΔSC: *β* = −0.019, *SE *= 0.113, *z *= −0.17, *P* = .864; alexithymia × valence: *β* = −0.031, *SE *= 0.046, *z *= −0.67, *P* = .500; alexithymia × arousal: *β*  =  0.010, *SE *= 0.062, *z *= 0.15, *P* = .877; ΔSC × valence: *β* = −0.059, *SE *= 0.044, *z *= −1.34, *P* = .179; ΔSC × arousal: *β* = −0.070, *SE *= 0.060, *z *= −1.16, *P* = .248). In other words, individual differences in alexithymia and self-construal did not significantly influence decisions about whether to express or suppress emotion.

As an exploratory analysis, we tested possible nonlinear effects of valence and arousal. Regression analysis, including quadratic terms revealed a significant quadratic effect of valence (*β*  =  0.571, *SE *= 0.054, *z *= 10.51, *P* < .001), indicating a U-shaped relationship in which both highly positive and highly negative situations were more likely to elicit emotional expression than neutral ones. The quadratic effect of arousal was not significant (*β*  =  0.032, *SE *= 0.036, *z *= 0.89, *P* = .371). An additional analysis further assessed whether emotional expression decisions varied across different types of social contexts: i.e. relational and individual scenarios. We found a marginally significant effect of context (*β*  =  0.21, *SE *= 0.11, *z *= 1.83, *P* = .067), suggesting a trend toward increased expression in relational scenarios. We also examined whether the effects of valence and arousal differ between engaged and disengaged scenarios. Engagement showed a significant positive association with emotional expression (*β*  =  0.617, *SE *= 0.085, *t *= 7.23, *P* < .001). The interaction between engagement and valence was also significant (*β*  =  0.219, *SE *= 0.054, *t *= 4.03, *P* < .001), whereas the interaction between engagement and arousal was not (*β* = −0.072, *SE *= 0.052, *t *= −1.38, *P* = .166). These findings suggest that the influence of valence on decisions to express or suppress emotions differed between engaged and disengaged scenarios. Specifically, the association between valence and emotional expression decisions was stronger for scenarios involving engaged emotions than for those involving disengaged emotions. In contrast, the influence of arousal did not differ between the two scenario types.

### Neural correlates: emotion vs. control condition

**Figure 3:**
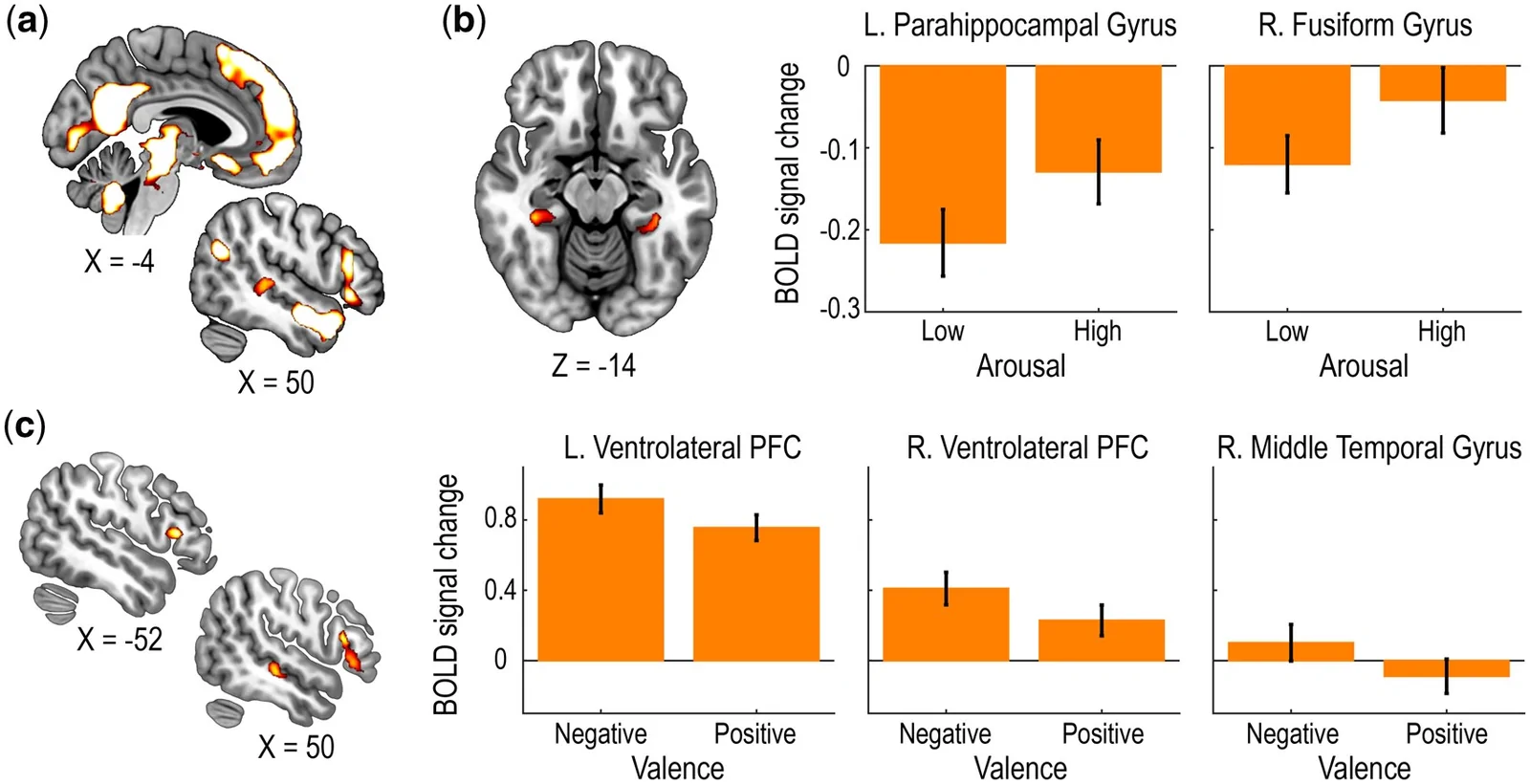
Neural correlates of emotion expression decision-making, perceived arousal, and valence. (a) Neural correlates of emotion expression decision-making. Statistical map showing neural activity associated with the contrast between the Emotion and Control conditions. The activation map is thresholded at *P* < .001 (uncorrected) for display purposes. (b) Neural correlates of perceived arousal in the Emotion condition. Statistical map showing neural activity correlated with perceived arousal in the Emotion condition. The activation map is thresholded at *P* < .001 (uncorrected) for display purposes. Bar plots show changes in the BOLD signal separately for low- and high-arousal scenarios for display purposes. (c) Neural correlates of perceived valence in the Emotion condition. Statistical map showing neural activity negatively correlated with perceived valence in the Emotion condition. The format is the same as in (b).

Comparison between the Emotion and Control conditions revealed differential activity in mentalizing-related brain regions, including the superior frontal gyrus, precuneus/posterior cingulate cortex, and temporoparietal junction (TPJ) (*P* < .05, cluster-level corrected; see Fig. 3a and Table 2), consistent with previous findings (e.g. Saxe and Kanwisher 2003, Saxe and Wexler 2005). Specifically, BOLD signal in these regions was greater in the Emotion condition than in the Control condition at the time of scenario reading.

**Table 1:** Participant characteristics.

Variable	Mean (SD)/*N*
Sample size	40
Age (years)	21.1 (2.2)
Sex (male/female)	22/18
TAS-20 score	49.68 (8.88)
SC (independent)	4.73 (0.83)
SC (interdependent)	4.99 (0.67)

**Table 2:** Whole-brain peak coordinates.

Contrast	Region	Hemi	x	y	z	*t*-value	Cluster size
Emotion > Control	Lateral orbital gyrus (vlPFC)	L	−43	28	−13	13.71	8114
	Precuneus	L/R	7	−53	23	11.14	4976
	Lateral orbital gyrus (vlPFC)	R	41	33	−18	10.07	1851
	Cerebellum	L/R	0	−55	−35	9.79	612
	Middle occipital gyrus/angular gyrus/supramarginal gyrus (TPJ)	L	−50	−70	23	8.25	802
	Angular gyrus (TPJ)	R	50	−63	25	6.56	223
	Inferior parietal lobule	R	29	−36	25	5.14	201
	Superior temporal gyrus	R	45	−34	4	4.84	132
	Inferior occipital gyrus	R	29	−89	−1	4.75	364
Valence (negative association)	Inferior frontal gyrus (IFG; vlPFC)	R	50	28	−4	5.11	221
	Inferior frontal gyrus (IFG; vlPFC)	L	−50	24	8	5.06	125
	Middle temporal gyrus (MTG)/superior temporal sulcus (STS)	R	62	−15	−11	4.60	143
Arousal (positive association)	Fusiform gyrus (FG)	R	38	−34	−16	4.79	132
	Parahippocampal gyrus (PHG)	L	−36	−34	−13	4.62	118
Emotion condition: No > Yes	Angular gyrus (TPJ)	R	60	−58	35	6.82	267
	Middle frontal gyrus (dlPFC)	R	48	21	44	4.76	148
	Superior frontal gyrus	R	19	24	61	4.93	209
Valence (Emotion > Control)	Intraparietal sulcus (IPS)/dorsal inferior parietal lobule (IPL)	R	31	−41	35	5.10	230

Note. Coordinates are reported in MNI space. Statistical maps were thresholded at *P* < .001 (uncorrected) with cluster-level family-wise error (FWE) correction at *P* < .05. Hemi, hemisphere.

### Neural correlates: arousal and valence in the emotion condition

Perceived arousal was associated with increased activity in the left parahippocampal gyrus (PHG) and right fusiform gyrus (FG) (*P* < .05, cluster-level corrected; Fig. 3b and Table 2). BOLD signal levels were positively correlated with arousal ratings during scenario reading. Valence was negatively associated with activity in the bilateral vlPFC and right middle temporal gyrus (MTG) (*P* < .05, cluster-level corrected; see Fig. 3c and Table 2), such that these regions showed greater activation for more negatively valenced (i.e. more unpleasant) scenarios.

### Neural correlates: No vs. yes response in the emotion condition

**Figure 4:**
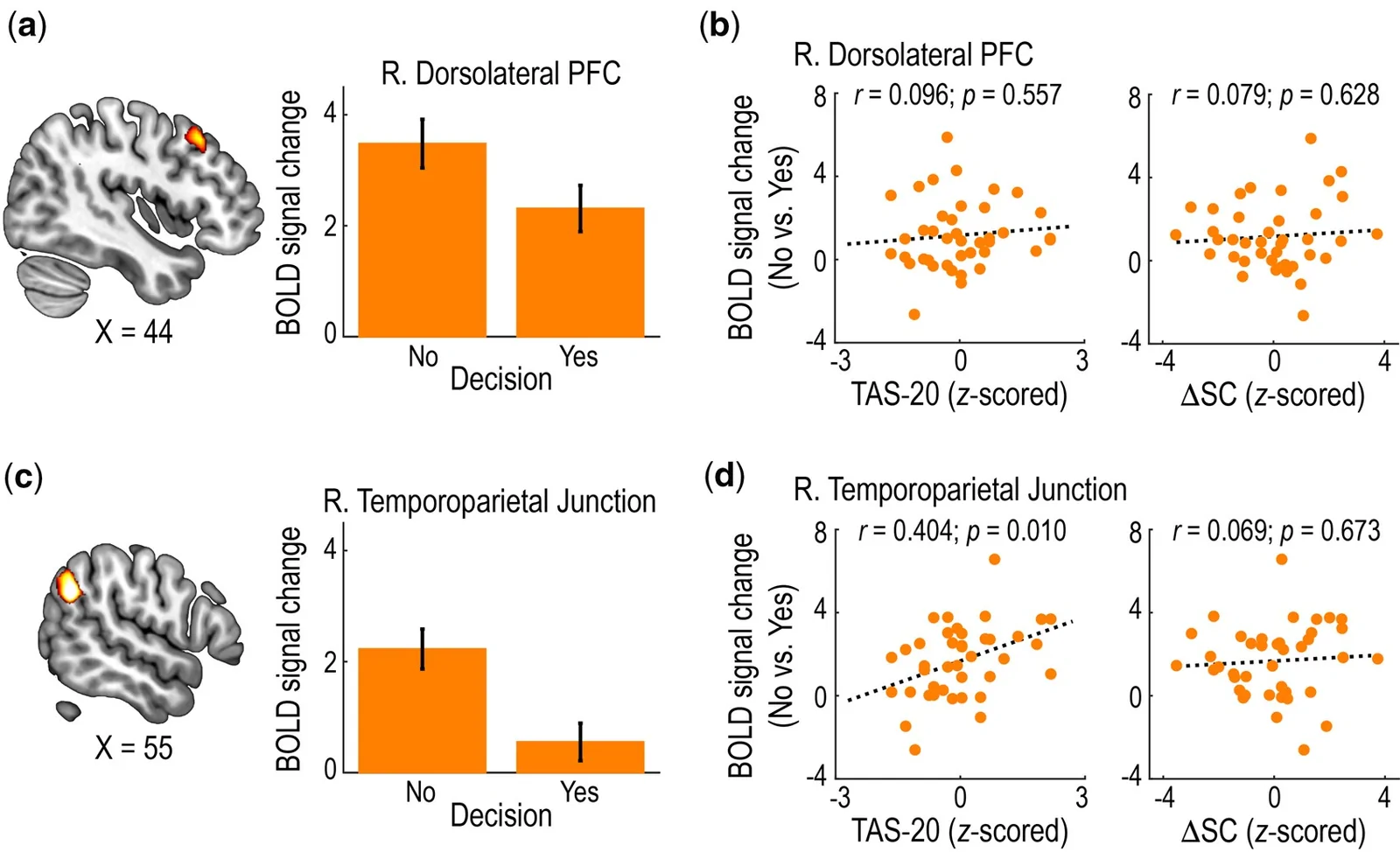
Neural correlates of decisions to suppress emotional expression. (a) Neural correlates of decisions to withhold emotion expression in the Emotion condition in the right dorsolateral prefrontal cortex (dlPFC). Statistical map showing neural activity associated with the contrast of No > Yes decisions in the Emotion condition. The activation map is thresholded at *P* < .001 (uncorrected) for display purposes. Bar plots show BOLD signal changes for display purposes. (b) Across-participant correlations between decision-related neural activity in the right dorsolateral prefrontal cortex (dlPFC) and questionnaire scores. Left, neural activity is plotted as a function of scores on the 20-item Toronto Alexithymia Scale (TAS-20). Right, neural activity is plotted as a function of the difference between the interdependent and independent subscale scores of the SCS (ΔSC; interdependent minus independent). (c) Neural correlates of decisions to withhold emotion expression in the Emotion condition in the right temporoparietal junction. The format is the same as in (a). (d) Across-participant correlations between decision-related neural activity in the right temporoparietal junction and questionnaire scores. The format is the same as in (b).

Decisions to suppress emotional expression (No > Yes responses) were associated with increased activation in the right TPJ and the right dlPFC (*P* < .05, cluster-level corrected; Fig. 4a and c and Table 2). Specifically, BOLD signal in the decision phase was significantly higher for No responses than for Yes responses. Finally, we examined whether decision-related neural activity in the TPJ and dlPFC was associated with individual differences in alexithymia and the self-construal difference score (ΔSC) (Fig. 4b and d). We found a significant across-participant correlation between alexithymia questionnaire scores and neural activity in the right TPJ during decisions to suppress, rather than express, emotional responses (Fig. 4d; *r* = .404, *P* = .010). This correlation remained significant after correction for multiple comparisons (*P* = .040).

## Discussion

The present study elucidates the behavioral and neural mechanisms underlying decisions about emotional expression. By combining behavioral modeling with fMRI, we examined whether and how emotional responses are expressed or suppressed in a context-dependent manner, as well as the neural mechanisms underlying these decisions.

Behaviorally, we demonstrated that decisions about whether to express or suppress emotion depend on context. Specifically, participants were more likely to express their emotions when the perceived valence and arousal of the emotional situation were high, suggesting that these dimensions serve as key inputs to the decision process. This interpretation is consistent with recent work suggesting that value computations underpin flexible emotional expression (Teoh and Hutcherson 2025). More broadly, this finding challenges the common view that emotional expression is an automatic, spontaneous consequence of affective experience (Ekman 1992, Izard 1997). Instead, it supports the alternative view that emotional expression results from a decision-making process in which affective information is evaluated in light of anticipated interpersonal consequences (Frijda 1986, Parkinson 1996, Van Kleef 2009, Lockwood *et al.* 2020).

Perceived valence and arousal were associated with activity in the bilateral vlPFC and right MTG, and in the left PHG and the right FG, respectively. Notably, activity in the vlPFC and MTG showed a negative correlation with perceived valence, which may reflect greater demands on evaluative, regulatory, and contextual processing in negative scenarios. Negative situations often involve potential social conflict, norm violations, or interpersonal risk (Baumeister *et al.* 2001, Van Kleef 2009) and may therefore require more elaborate evaluation and response selection. This effect may have been particularly pronounced in the present study because many scenarios involved socially regulated emotions, such as guilt or shame. These emotions, often characterized as interpersonally engaged (Kitayama *et al.* 2000), may place greater demands on behavioral control and context-sensitive evaluation, thereby recruiting vlPFC and MTG processes implicated in inhibition, semantic retrieval, and situation comprehension (Badre and Wagner 2007, Binder *et al.* 2009, Lambon Ralph *et al.* 2017, Satpute and Lindquist 2019). By contrast, activity in the PHG/FG associated with perceived arousal may reflect the construction of situational context and mental imagery, supporting the integration of complex visual and contextual features that contribute to emotional experience (Kragel *et al.* 2019).

While prior studies using perceptually salient emotional stimuli such as facial expressions or aversive images have consistently implicated limbic and paralimbic regions in affective processing (Kober *et al.* 2008, Pessoa 2008, Lindquist *et al.* 2012, Satpute and Lindquist 2019, Schurz *et al.* 2021), the present study primarily engaged regions involved in semantic and contextual processing, including the bilateral vlPFC, right MTG, and bilateral PFG/FG. This difference likely reflects the use of scenario-based linguistic stimuli, which required participants to construct the meaning of the situation and infer its emotional significance. The MTG may support semantic processing of scenario content, the vlPFC may evaluate its contextual and social relevance, and the PHG/FG may contribute to situational context construction and mental imagery. Together, these regions may support the integration of semantic understanding and social evaluation required for decisions about whether to express emotion.

Recent perspectives further suggest that valence and arousal are not represented in a one-to-one manner in specific brain regions but rather emerge from distributed and context-dependent neural processes (Skerry and Saxe 2015, Riberto *et al.* 2022). In this context, the involvement of vlPFC, MTG, and PHG/FG observed in the present study is consistent with the idea that affective information is constructed through semantic and contextual integration rather than driven by immediate sensory-affective responses (Riberto *et al.* 2022). This may reflect a greater reliance on inferred social and contextual cues, rather than direct access to one’s own internal affective states, when making decisions about emotional expression.

Decisions to suppress emotional expression were associated with neural activity in the right TPJ and dlPFC. The TPJ has been consistently implicated in social cognition and mentalizing, particularly in representing others’ beliefs and intentions (Saxe and Kanwisher 2003, Frith and Frith 2006, Buergi *et al.* 2026), whereas the dlPFC has been linked to cognitive control and the inhibition of prepotent responses (Miller and Cohen 2001, Ochsner and Gross 2005, Morawetz *et al.* 2020). Previous findings further indicate a right-lateralized contribution to perspective-taking and social cognition (Mitchell 2008). Together, these findings suggest that TPJ and dlPFC support the suppression of emotional expression through the integration of social inference and cognitive control processes (Ochsner and Gross 2005, Morawetz *et al.* 2020, Sikka *et al.* 2022). Consistent with prior work on expressive suppression, which has been associated with increased activation in frontoparietal control regions, including the lateral prefrontal cortex and inferior parietal cortex (Sikka *et al.* 2022), the present findings suggest that decisions to suppress emotional expression recruit domain-general cognitive control mechanisms. However, whereas prior studies have primarily examined suppression as a regulatory response to externally presented emotional stimuli, the present findings suggest that similar frontoparietal mechanisms may also be engaged at an earlier decision stage, in which individuals determine whether emotional responses should be expressed.

Building on these findings, we suggest that decisions about emotional expression may arise from interactions among systems involved in sensory processing, semantic integration, social inference, and cognitive control. In the present paradigm, written scenarios are likely first processed by the visual system, with early visual areas (e.g. V1) extracting orthographic information, followed by processing along the ventral visual stream, including regions such as the FG that are involved in word and form recognition (Binder *et al.* 2009, Dehaene and Cohen 2011, Satpute and Lindquist 2019). Semantic and contextual information may then be integrated in temporal regions, including the MTG and anterior temporal lobe, which have been implicated in language comprehension and conceptual processing. These representations may in turn provide input to higher-order social cognitive systems, including the TPJ, which is known to support mentalizing and the representation of others’ mental states (Saxe and Kanwisher 2003, Buergi *et al.* 2026). This interpretation is consistent with prior work showing that narrative-based perspective-taking recruits the TPJ even in the absence of explicit social interaction (Mano *et al.* 2009, Miao *et al.* 2026). Finally, the dlPFC may contribute to the selection of goal-directed responses by integrating socially inferred consequences with current task demands (Ochsner and Gross 2005) and has also been shown to play a causal role in guiding advantageous choices under uncertainty (Morawetz *et al.* 2020, Obeso *et al.* 2021). This flow of information—from perceptual and semantic analysis to social inference and executive control—may underlie decisions about whether to express emotion in a given social context. This account is consistent with recent integrative frameworks of social cognition and emotion regulation, such as the Cognitive–Affective Social Processing and Emotion Regulation (CASPER) model, which proposes that social behavior emerges from dynamic interactions among perceptual, interpretive, and regulatory processes shaped by affect and goals (Camacho *et al.* 2026).

Furthermore, we found that individual differences in alexithymia were associated with decision-related neural activity in the right TPJ, although they were not correlated with behavioral expression decisions. This dissociation suggests that alexithymia may influence the neural strategies underlying emotional expression decisions rather than overt behavior itself. One possibility is that individuals with higher alexithymia rely more strongly on controlled social inference processes when making expression decisions. However, the absence of behavioral effects should be interpreted with caution, and future studies with larger samples will be needed to clarify the role of individual differences.

We did not observe significant associations between self-construal and behavioral or neural measures across participants. While null findings are hard to interpret, in the present case, the decision to express or suppress emotions may depend on the perceived desirability of these emotions (Markus and Kitayama 1991). In particular, cultural frameworks such as affect valuation theory propose that the desirability of emotional states varies across cultural contexts (Tsai *et al.* 2006), which may influence decisions to express or suppress emotions. The perceived desirability may depend largely on social norms in different cultures and therefore it may be largely shared regardless of individual differences in self-construal. Further, from this perspective, the neural mechanisms identified in the present study—particularly the involvement of TPJ and its interaction with dlPFC—may not be universal but instead may be modulated by culturally shaped expectations about emotional expression and social harmony. For example, individuals from interdependent cultural contexts may place greater emphasis on anticipating others’ reactions, potentially increasing reliance on mentalizing processes during expression decisions. Future studies using larger and more diverse samples, including direct cross-cultural comparisons (e.g. Japanese and U.S. populations), will be essential for determining whether the present findings generalize across cultures or show systematic cultural modulation.

Several limitations should be noted. First, the sample size limits statistical power for detecting subtle individual differences. Second, the use of scenario-based stimuli may not fully capture the complexity of real-world emotional interactions. In everyday social situations, emotional expression unfolds dynamically in response to facial expressions, vocal tone, and ongoing feedback from others, whereas the present task required participants to evaluate written scenarios in a relatively constrained and decontextualized manner. Although this design allowed precise experimental control, it may have reduced ecological validity. Future studies using more naturalistic and interactive paradigms will be important for clarifying how emotional expression decisions operate in real-world social settings. Third, affective dimensions were assessed using post-scan self-reports, which may not fully reflect moment-to-moment emotional experiences during the decision-making process. Future studies combining online measures or physiological indices may help to more precisely capture affective dynamics. Fourth, the control condition required less semantic and emotional processing than the emotion condition. Therefore, some of the observed differences between conditions may reflect differences in cognitive demands rather than emotional processing alone. Moreover, the greater activity observed during suppression (“No”) decisions should not necessarily be interpreted as reflecting suppression processes per se. An alternative explanation is that choosing not to express emotion requires greater cognitive effort or decision uncertainty than choosing to express emotion. Other factors, such as differences in reaction time, response ambiguity, or the everyday familiarity of the scenarios, may also have contributed to the observed neural activity. Future studies should incorporate control conditions matched for semantic processing demands and explicitly evaluate these factors to better distinguish neural activity associated with emotion regulation from more general decision-related processes. Finally, although the present study focused on valence and arousal as key inputs to decision-making, emotional expression in real-world contexts may be influenced by additional factors such as social norms, interpersonal relationships, and situational constraints. Future work will be needed to examine how these factors are integrated within the decision process. Integrating large-scale behavioral datasets and computational modeling approaches (e.g. Lockwood *et al.* 2020, Suzuki and O’Doherty 2020) may further refine our understanding of emotional expression decisions.

The present findings may also have implications for theories of emotion regulation. Traditional models have primarily focused on how emotional responses are modulated after they are generated (Gross 1998, 2002, Ochsner and Gross 2005, Buhle *et al.* 2014, Ford and Gross 2019, Morawetz *et al.* 2020). More recent frameworks, however, conceptualize emotion regulation as a multi-stage process involving identification, selection, and implementation of regulatory strategies (Sikka *et al.* 2022, Liu *et al.* 2023). Within this broader perspective, regulation is not limited to post-response modulation but may also involve anticipatory and decision-related processes. The present results extend this view by suggesting that emotional expression involves an evaluative stage, in which individuals determine whether emotional responses should be expressed. Rather than replacing existing models, this finding points to an upstream decision process that shapes whether regulatory mechanisms are engaged. This perspective highlights emotional expression as an integral component of emotion regulation, embedded within value-based decision-making in social contexts, and may also be relevant to sociocultural approaches emphasizing interpersonal and cultural influences (Ma *et al.* 2018).

In conclusion, the present study provides important insights into how individuals choose to withhold emotional expression. Our findings suggest that the suppression of emotional expression may arise from a decision-making process implemented in brain regions implicated in mentalizing and cognitive control, including the right temporoparietal junction and dorsolateral prefrontal cortex. More broadly, the present study highlights a novel aspect of affective neuroscience: decision-making about whether to express emotion.

## Supplementary Material

nsag068_Supplementary_Data

## Data Availability

The data that support the findings of this study are publicly available on OpenNeuro at https://doi.org/10.18112/openneuro.ds008583.v1.0.0. Analysis code associated with this study is available at https://github.com/yokomano/Emotion-Expression-fMRI.

## References

[nsag068-B1] Badre D , Wagner AD. Left ventrolateral prefrontal cortex and the cognitive control of memory. Neuropsychologia 2007;45:2883–901. 10.1016/j.neuropsychologia.2007.06.015 17675110

[nsag068-B2] Bagby RM , Parker JDA, Taylor GJ. The twenty-item Toronto Alexithymia Scale—I. Item selection and cross-validation of the factor structure. J Psychosom Res 1994;38:23–32. 10.1016/0022-3999(94)90005-1 8126686

[nsag068-B3] Barrett LF. How Emotions are Made: The Secret Life of the Brain. Boston: Houghton Mifflin Harcourt, 2017. https://psycnet.apa.org/record/2017-26294-000

[nsag068-B4] Barrett LF , Russell JA. The structure of current affect: controversies and emerging consensus. Curr Dir Psychol Sci 1999;8:10–4. 10.1111/1467-8721.00003

[nsag068-B5] Baumeister RF , Bratslavsky E, Finkenauer C et al Bad is stronger than good. Rev Gen Psychol 2001;5:323–70. 10.1037/1089-2680.5.4.323

[nsag068-B6] Binder JR , Desai RH, Graves WW et al Where is the semantic system? A critical review and meta-analysis of 120 functional neuroimaging studies. Cereb Cortex 2009;19:2767–96. 10.1093/cercor/bhp055 PMC2774390 19329570

[nsag068-B7] Bird G , Cook R. Mixed emotions: the contribution of alexithymia to the emotional symptoms of autism. Transl Psychiatry 2013;3:e285. 10.1038/tp.2013.61 PMC3731793 23880881

[nsag068-B8] Buhle JT , Silvers JA, Wager TD et al Cognitive reappraisal of emotion: a meta-analysis of human neuroimaging studies. Cereb Cortex 2014;24:2981–90. 10.1093/cercor/bht154 PMC4193464 23765157

[nsag068-B9] Buergi N , Aydogan G, Konovalov A et al A neural signature of adaptive mentalization. Nat Neurosci 2026;29:934–44. 10.1038/s41593-026-02219-x PMC13061600 41803318

[nsag068-B10] Camacho MC , Deshpande E, Perino MT. The cognitive–affective social processing and emotion regulation (CASPER) model. Neuropsychopharmacology 2026;51:136–52. 10.1038/s41386-025-02005-9 PMC12618608 40908288

[nsag068-B11] Dehaene S , Cohen L. The unique role of the visual word form area in reading. Trends Cogn Sci 2011;15:254–62. 10.1016/j.tics.2011.04.003 21592844

[nsag068-B12] Eekhof LS , Van Krieken K, Willems RM. Reading about minds: the social-cognitive potential of narratives. Psychon Bull Rev 2022;29:1703–18. 10.3758/s13423-022-02079-z PMC9568452 35318585

[nsag068-B13] Ekman P. An argument for basic emotions. Cognition & Emotion 1992;6:169–200. 10.1080/02699939208411068

[nsag068-B14] Esteban O , Markiewicz CJ, Blair RW et al fMRIPrep: a robust preprocessing pipeline for functional MRI. Nat Methods 2019;16:111–6. 10.1038/s41592-018-0235-4 PMC6319393 30532080

[nsag068-B15] Ford BQ , Gross JJ. Why beliefs about emotion matter: an emotion-regulation perspective. Curr Dir Psychol Sci 2019;28:74–81. 10.1177/0963721418806697

[nsag068-B16] Frijda NH. The Emotions. Cambridge: Cambridge University Press, 1986. https://psycnet.apa.org/record/1987-97938-000

[nsag068-B17] Frith CD , Frith U. The neural basis of mentalizing. Neuron 2006;50:531–4. 10.1016/j.neuron.2006.05.001 16701204

[nsag068-B18] Gross JJ. The emerging field of emotion regulation: an integrative review. Rev Gen Psychol 1998;2:271–99. 10.1037/1089-2680.2.3.271

[nsag068-B19] Gross JJ. Emotion regulation: affective, cognitive, and social consequences. Psychophysiology 2002;39:281–91. 10.1017/S0048577201393198 12212647

[nsag068-B20] Izard CE. Human Emotions. New York: Springer, 1997. 10.1007/978-1-4899-2209-0

[nsag068-B21] Keltner D , Haidt J. Social functions of emotions at four levels of analysis. Cogn Emot 1999;13:505–21. 10.1080/026999399379168

[nsag068-B22] Kitayama S , Markus HR, Kurokawa M. Culture, emotion, and well-being: Good feelings in Japan and the United States. Cogn Emot 2000;14:93–124. 10.1080/026999300379003

[nsag068-B23] Kober H , Barrett LF, Joseph J et al Functional grouping and cortical-subcortical interactions in emotion: a meta-analysis of neuroimaging studies. Neuroimage 2008;42:998–1031. 10.1016/j.neuroimage.2008.03.059 PMC2752702 18579414

[nsag068-B24] Komeda H , Kawasaki M, Tsunemi K et al Differences between estimating protagonists’ emotions and evaluating readers’ emotions in narrative comprehension. Cogn Emot 2009;23:135–51. 10.1080/02699930801949116

[nsag068-B25] Kragel PA , Reddan MC, LaBar KS et al Emotion schemas are embedded in the human visual system. Sci Adv 2019;5:eaaw4358. 10.1126/sciadv.aaw4358 PMC6656543 31355334

[nsag068-B26] Kuppens P , Champagne D, Tuerlinckx F. The dynamic interplay between appraisal and core affect in daily life. Front Psychol 2012;3:380. 10.3389/fpsyg.2012.00380 PMC3466066 23060842

[nsag068-B27] Kuppens P , Tuerlinckx F, Yik M et al The relation between valence and arousal in subjective experience varies with personality and culture. J Pers 2017;85:530–42. 10.1111/jopy.12258 27102867

[nsag068-B28] Lambon Ralph MA , Jefferies E, Patterson K et al The neural and computational bases of semantic cognition. Nat Rev Neurosci 2017;18:42–55. 10.1038/nrn.2016.150 27881854

[nsag068-B29] Lieberman MD , Eisenberger NI, Crockett MJ et al Putting feelings into words: Affect labeling disrupts amygdala activity in response to affective stimuli. Psychol Sci 2007;18:421–8. 10.1111/j.1467-9280.2007.01916.x 17576282

[nsag068-B30] Lindquist KA , Wager TD, Kober H et al The brain basis of emotion: a meta-analytic review. Behav Brain Sci 2012;35:121–43. 10.1017/S0140525X11000446 PMC4329228 22617651

[nsag068-B31] Liu Z , Lu K, Hao N et al Cognitive reappraisal and expressive suppression evoke distinct neural connections during interpersonal emotion regulation. J Neurosci 2023;43:8456–71. 10.1523/JNEUROSCI.0954-23.2023 PMC10711701 37852791

[nsag068-B32] Lockwood PL , Apps MAJ, Chang SWC. Is there a “social” brain? Implementations and algorithms. Trends Cogn Sci 2020;24:802–13. 10.1016/j.tics.2020.06.011 PMC7501252 32736965

[nsag068-B33] Ma X , Tamir M, Miyamoto Y. Socio-cultural instrumental approach to emotion regulation: Culture and the regulation of positive emotions. Emotion 2018;18:138–52. 10.1037/emo0000337 28414476

[nsag068-B34] Mano Y , Harada T, Sugiura M et al Perspective-taking as part of narrative comprehension: a functional MRI study. Neuropsychologia 2009;47:813–24. 10.1016/j.neuropsychologia.2008.12.011 19135072

[nsag068-B35] Markus HR , Kitayama S. Culture and the self: implications for cognition, emotion, and motivation. Psychol Rev 1991;98:224–53. 10.1037/0033-295X.98.2.224

[nsag068-B36] Miao Z , Jung H, Kragel PA et al Common and distinct neural correlates of social interaction processing and theory of mind in narratives. Nat Commun 2026;17:4830. 10.1038/s41467-026-71151-2 PMC13223265 41935085

[nsag068-B37] Miller EK , Cohen JD. An integrative theory of prefrontal cortex function. Annu Rev Neurosci 2001;24:167–202. 10.1146/annurev.neuro.24.1.167 11283309

[nsag068-B38] Mitchell JP. Activity in right temporo-parietal junction is not selective for theory-of-mind. Cereb Cortex 2008;18:262–71. 10.1093/cercor/bhm051 17551089

[nsag068-B41] Morawetz C , Riedel MC, Salo T et al Multiple large-scale neural networks underlying emotion regulation. Neurosci Biobehav Rev 2020;116:382–95. 10.1016/j.neubiorev.2020.07.001 32659287

[nsag068-B42] Obeso I , Herrero M-T, Ligneul R et al A causal role for the right dorsolateral prefrontal cortex in avoidance of risky choices and making advantageous selections. Neuroscience 2021;458:166–79. 10.1016/j.neuroscience.2020.12.035 33476698

[nsag068-B43] Ochsner KN , Gross JJ. The cognitive control of emotion. Trends Cogn Sci 2005;9:242–9. 10.1016/j.tics.2005.03.010 15866151

[nsag068-B44] Ohnishi K , Sugawara M, Mano Y et al Neural mechanisms underlying reward processing and social cognition: a replication study with a Japanese sample. PLoS One 2025;20:e0328424. 10.1371/journal.pone.0328424 PMC12543148 41124148

[nsag068-B45] Parkinson B. Emotions are social. Br J Psychol 1996;87 ( Pt 4):663–83. 10.1111/j.2044-8295.1996.tb02615.x 8962482

[nsag068-B46] Pessoa L. On the relationship between emotion and cognition. Nat Rev Neurosci 2008;9:148–58. 10.1038/nrn2317 18209732

[nsag068-B47] Riberto M , Paz R, Pobric G et al The neural representations of emotional experiences are more similar than those of neutral experiences. J Neurosci 2022;42:2772–85. 10.1523/JNEUROSCI.1490-21.2022 PMC8973424 35165174

[nsag068-B48] Russell JA. A circumplex model of affect. J Pers Soc Psychol 1980;39:1161–78. 10.1037/h0077714

[nsag068-B49] Sabag-Shushan T , Katzir T, Kanat-Maymon Y. The contribution of emotion vocabulary to the reading comprehension of the text and the task. Read Writ 2025;38:2609–32. 10.1007/s11145-024-10609-5

[nsag068-B50] Satpute AB , Lindquist KA. The default mode network’s role in discrete emotion. Trends Cogn Sci 2019;23:851–64. 10.1016/j.tics.2019.07.003 PMC7281778 31427147

[nsag068-B51] Saxe R , Kanwisher N. People thinking about thinking people: the role of the temporo-parietal junction in “theory of mind”. Neuroimage 2003;19:1835–42. 10.1016/S1053-8119(03)00230-1 12948738

[nsag068-B52] Saxe R , Wexler A. Making sense of another mind: the role of the right temporo-parietal junction. Neuropsychologia 2005;43:1391–9. 10.1016/j.neuropsychologia.2005.02.013 15936784

[nsag068-B53] Schurz M , Radua J, Tholen MG et al Toward a hierarchical model of social cognition: a neuroimaging meta-analysis and integrative review. Psychol Bull 2021;147:293–327. 10.1037/bul0000303 33151703

[nsag068-B54] Sikka P , Stenberg J, Vorobyev V et al The neural bases of expressive suppression: a systematic review of functional neuroimaging studies. Neurosci Biobehav Rev 2022;138:104708. 10.1016/j.neubiorev.2022.104708 35636561

[nsag068-B55] Singelis TM. The measurement of independent and interdependent self-construals. Pers Soc Psychol Bull 1994;20:580–91. 10.1177/0146167294205014

[nsag068-B56] Skerry AE , Saxe R. Neural representations of emotion are organized around abstract event features. Curr Biol 2015;25:1945–54. 10.1016/j.cub.2015.06.009 PMC4824044 26212878

[nsag068-B57] Smith R , Lane RD, Alkozei A et al Maintaining the feelings of others in working memory is associated with activation of the left anterior insula and left frontal-parietal control network. Soc Cogn Affect Neurosci 2017;12:848–60. 10.1093/scan/nsx011 PMC5460045 28158779

[nsag068-B58] Suzuki S , O’Doherty JP. Breaking human social decision making into multiple components and then putting them together again. Cortex 2020;127:221–30. 10.1016/j.cortex.2020.02.014 PMC7751985 32224320

[nsag068-B59] Taylor GJ , Bagby RM, Parker JDA. The 20-item toronto alexithymia scale—IV. Reliability and factorial validity in different languages and cultures. J Psychosom Res 2003;55:277–83. 10.1016/S0022-3999(02)00601-3 12932803

[nsag068-B60] Teoh YY , Hutcherson CA. Value computations underpin flexible emotion expression. Commun Psychol 2025;3:169. 10.1038/s44271-025-00169-x PMC12643929 41286359

[nsag068-B61] Tsai JL , Knutson B, Fung HH. Cultural variation in affect valuation. J Pers Soc Psychol 2006;90:288–307. 10.1037/0022-3514.90.2.288 16536652

[nsag068-B62] Van Kleef GA. How emotions regulate social life: the emotions as social information (EASI) model. Curr Dir Psychol Sci 2009;18:184–8. 10.1111/j.1467-8721.2009.01633.x

[nsag068-B63] Van Overwalle F. Social cognition and the brain: a meta-analysis. Hum Brain Mapp 2009;30:829–58. 10.1002/hbm.20547 Participant characteristics PMC6870808 18381770

