# Supplementary material for: Neural mechanisms underlying decisions to express or suppress emotions
Source: Soc Cogn Affect Neurosci. 2026 Aug 7;21(1):nsag068. doi: 10.1093/scan/nsag068 (PMC13568820; doi:10.1093/scan/nsag068)
Supplement: Supplementary file 1 [file nsag068_supplementary_data.zip › Supplementary Table S1_rev1.docx]

| Supplementary Table S1. Emotional sentence stimuli categorized by emotion (Kitayama, Markus, & Kurokawa, 2000) | | |
| --- | --- | --- |
| **Emotion category (Kitayama, Markus, & Kurokawa, 2000)** | **Scenario sentences** | **Context type** |
| Superior | I was studying with a close friend and was the only one who understood, so I felt superior. | relational |
| (Disengaged) | I was the only one in my family who could use a new technology well, so I felt superior. | relational |
|  | I solved a difficult math problem faster than an acquaintance, so I felt superior. | relational |
|  | I was able to demonstrate more knowledge than someone I had just met, so I felt superior. | relational |
|  | I quickly solved a difficult statistics problem on my own, so I felt superior. | individual |
|  | I realized again how high my problem-solving ability was, so I felt superior. | individual |
|  | My opinion was the only one supported in an important meeting, so I felt superior. | relational |
|  | I attracted a lot of attention at an event, so I felt superior. | relational |
| Proud | My close friend highly praised my idea, so I felt proud. | relational |
| (Disengaged) | My family praised my efforts so far, so I felt proud. | relational |
|  | An acquaintance praised my deep expertise, so I felt proud. | relational |
|  | Someone I had just met said they respected me as an admirable person, so I felt proud. | relational |
|  | I achieved a difficult goal through trial and error on my own, so I felt proud. | individual |
|  | My efforts in practicing for a research presentation paid off, so I felt proud. | individual |
|  | My presentation succeeded in an important meeting and received praise, so I felt proud. | relational |
|  | I received high praise from the audience at a lecture, so I felt proud. | relational |
| **Emotion category (Kitayama, Markus, & Kurokawa, 2000)** | **Scenario sentences** | **Context type** |
| Top of the world | My close friend always did what I said, so I felt on top of the world. | relational |
| (Disengaged) | My family repeatedly relied on me, so I felt on top of the world. | relational |
|  | An acquaintance praised me excessively, so I felt on top of the world. | relational |
|  | An acquaintance bowed only to me, so I felt on top of the world. | relational |
|  | I succeeded repeatedly on my own, so I felt on top of the world. | individual |
|  | My work progressed without anyone pointing out mistakes, so I felt on top of the world. | individual |
|  | My opinion was adopted in a meeting, so I felt on top of the world. | relational |
|  | I was treated as the center of attention at an event, so I felt on top of the world. | relational |
| Anger | My close friend forgot an important anniversary, so I felt angry. | relational |
| (Disengaged) | My family ignored what I said at dinner, so I felt angry. | relational |
|  | Someone I had just met treated me rudely, so I felt angry. | relational |
|  | An acquaintance made an unreasonable criticism toward me, so I felt angry. | relational |
|  | Something important broke while I was working alone, so I felt angry. | individual |
|  | I remembered a past failure in a quiet room, so I felt angry. | individual |
|  | I was pushed on a crowded train platform, so I felt angry. | relational |
|  | My opinion was interrupted in a meeting I had prepared for, so I felt angry. | relational |
|  |  |  |
| **Emotion category (Kitayama, Markus, & Kurokawa, 2000)** | **Scenario sentences** | **Context type** |
| Frustration | My close friend repeatedly broke important promises, so I felt frustrated. | relational |
| (Disengaged) | My family did not accept my opinion, so I felt frustrated. | relational |
|  | An acquaintance repeatedly interrupted me, so I felt frustrated. | relational |
|  | A conversation proceeded while I was being misunderstood by someone I had just met, so I felt frustrated. | relational |
|  | I worked hard alone but saw no results, so I felt frustrated. | individual |
|  | Things did not go as I wanted despite repeated attempts, so I felt frustrated. | individual |
|  | I was not given any opportunity to speak in a meeting, so I felt frustrated. | relational |
|  | My role was undervalued at an event, so I felt frustrated. | relational |
| Sulky feelings | I was not invited while my close friend was, so I felt sulky. | relational |
| (Disengaged) | My family did not listen seriously to what I said, so I felt sulky. | relational |
|  | An acquaintance brushed off my suggestion, so I felt sulky. | relational |
|  | Someone I had just met ignored my greeting, so I felt sulky. | relational |
|  | No one acknowledged my efforts, so I felt sulky. | individual |
|  | Things did not go as I wanted and I became fed up with myself, so I felt sulky. | individual |
|  | My comments were repeatedly interrupted in a meeting, so I felt sulky. | relational |
|  | I was undervalued at an event, so I felt sulky. | relational |
|  |  |  |
| **Emotion category (Kitayama, Markus, & Kurokawa, 2000)** | **Scenario sentences** | **Context type** |
| Close | I shared my worries with a close friend, so I felt close. | relational |
| (Engaged) | I spent a long time with my family, so I felt close. | relational |
|  | I talked about a shared hobby with an acquaintance, so I felt close. | relational |
|  | I worked together with someone I had just met, so I felt close. | relational |
|  | I came across something nostalgic after a long time, so I felt close. | individual |
|  | I recalled memories with someone important, so I felt close. | individual |
|  | I got excited together with many people at a live event, so I felt close. | relational |
|  | I worked together with others in a community activity, so I felt close. | relational |
| Friendly feelings | I enjoyed a casual conversation with a close friend, so I felt friendly. | relational |
| (Engaged) | I looked back at old photos with my family, so I felt friendly. | relational |
|  | I had an engaging conversation about a shared hobby with an acquaintance, so I felt friendly. | relational |
|  | I laughed together with someone I had just met over a common topic, so I felt friendly. | relational |
|  | I remembered the voice of a friend after a long time, so I felt friendly. | individual |
|  | I recalled the kindness of someone important, so I felt friendly. | individual |
|  | I spent time pleasantly with people around me at an event, so I felt friendly. | relational |
|  | I naturally engaged in conversation at a community gathering, so I felt friendly. | relational |
|  |  |  |
| **Emotion category (Kitayama, Markus, & Kurokawa, 2000)** | **Scenario sentences** | **Context type** |
| Respect | My close friend overcame difficulties and achieved a goal, so I felt respect. | relational |
| (Engaged) | I saw my family treating others with sincerity, so I felt respect. | relational |
|  | An acquaintance behaved considerately toward others, so I felt respect. | relational |
|  | Someone I had just met confidently expressed their opinion, so I felt respect. | relational |
|  | I read about the life of a great person in a book, so I felt respect. | individual |
|  | I remembered unforgettable words from my mentor, so I felt respect. | individual |
|  | I listened to an inspiring talk at a lecture, so I felt respect. | relational |
|  | I saw a community leader guiding others, so I felt respect. | relational |
| Indebted | My close friend helped me many times when I was in trouble, so I felt indebted. | relational |
| (Engaged) | My family made sacrifices for me, so I felt indebted. | relational |
|  | An acquaintance prioritized helping me despite being busy, so I felt indebted. | relational |
|  | Someone I had just met treated me kindly, so I felt indebted. | relational |
|  | I recalled how many people had supported me in the past, so I felt indebted. | relational |
|  | I reflected alone on the kindness of others, so I felt indebted. | individual |
|  | People in my community cooperated for me, so I felt indebted. | relational |
|  | A colleague took over my work for me, so I felt indebted. | relational |
|  |  |  |
| **Emotion category (Kitayama, Markus, & Kurokawa, 2000)** | **Scenario sentences** | **Context type** |
| Ashamed | I forgot an important promise in front of my close friend, so I felt ashamed. | relational |
| (Engaged) | I made an inappropriate remark at a family gathering, so I felt ashamed. | relational |
|  | I gave incorrect information during a conversation with an acquaintance, so I felt ashamed. | relational |
|  | Someone I had just met ignored my greeting, so I felt ashamed. | relational |
|  | I recalled a past failure while alone, so I felt ashamed. | individual |
|  | I thought about stumbling over my words during a public presentation, so I felt ashamed. | individual |
|  | I failed to answer an important question in a meeting, so I felt ashamed. | relational |
|  | I fell and drew attention at an event, so I felt ashamed. | relational |
| Guilty | I failed to keep an important promise with my close friend, so I felt guilty. | relational |
| (Engaged) | I made an important decision without telling my family, so I felt guilty. | relational |
|  | I refused a request from an acquaintance, so I felt guilty. | relational |
|  | I treated someone I had just met coldly, so I felt guilty. | relational |
|  | I reflected on past mistakes while alone, so I felt guilty. | individual |
|  | I thought about causing trouble for others because of my actions, so I felt guilty. | individual |
|  | My comment caused problems for others in a meeting, so I felt guilty. | relational |
|  | I abandoned my role at an event, so I felt guilty. | relational |
|  |  |  |
